# Understanding Structural Features of Microbial Lipases—An Overview

**DOI:** 10.4137/aci.s551

**Published:** 2008-03-27

**Authors:** John Geraldine Sandana Mala, Satoru Takeuchi

**Affiliations:** 1 SANDANA FLORALS, Module-7, Golden Jubilee Biotech Park for Women Society, In SIPCOT-IT Park, Old Mahabalipuram Road, Siruseri, Navalur P.O., Kanchipuram District-603103, Tamilnadu, India; 2 Factory of Takeuchi Nenshi, TAKENEN, 85 NE, Takamatsu, Kahoku, Ishikawa 929-1215, Japan

**Keywords:** active site, bioinformatics, Candida rugosa lipase, crystallization, lipase structure, structure prediction

## Abstract

The structural elucidations of microbial lipases have been of prime interest since the 1980s. Knowledge of structural features plays an important role in designing and engineering lipases for specific purposes. Significant structural data have been presented for few microbial lipases, while, there is still a structure-deficit, that is, most lipase structures are yet to be resolved. A search for ‘lipase structure’ in the RCSB Protein Data Bank (http://www.rcsb.org/pdb/) returns only 93 hits (as of September 2007) and, the NCBI database (http://www.ncbi.nlm.nih.gov) reports 89 lipase structures as compared to 14719 core nucleotide records. It is therefore worthwhile to consider investigations on the structural analysis of microbial lipases. This review is intended to provide a collection of resources on the instrumental, chemical and bioinformatics approaches for structure analyses. X-ray crystallography is a versatile tool for the structural biochemists and is been exploited till today. The chemical methods of recent interests include molecular modeling and combinatorial designs. Bioinformatics has surged striking interests in protein structural analysis with the advent of innumerable tools. Furthermore, a literature platform of the structural elucidations so far investigated has been presented with detailed descriptions as applicable to microbial lipases. A case study of *Candida rugosa* lipase (CRL) has also been discussed which highlights important structural features also common to most lipases. A general profile of lipase has been vividly described with an overview of lipase research reviewed in the past.

## Introduction

Lipases (E.C.3.1.1.3) catalyze the hydrolysis of ester linkages in long-chain triacylglycerols with concomitant release of the constituent acid and alcohol moieties. They act at the interface between an insoluble substrate phase and an aqueous phase in which the enzyme is dissolved. Lipases are ubiquitously produced by plants (Bhardwaj et al. 2001), animals (Carriere et al. 1994) and microorganisms (Olempska-Beer et al. 2006). Microbial lipases are the preferred potent sources due to several industrial potentials (Hasan et al. 2006). The world market for lipases has been estimated at approximately US$20 million of the industrial enzyme market (Rahman et al. 2005). Lipases have been intensively investigated for their multiplexity of catalysis with unique specificities (Villeneuve and Foglia, 1997), which have multifold applications in oleochemistry, organic synthesis, detergent formulations and nutrition (Saxena et al. 2003). Also, lipases display useful properties related to their stability as organic solvent-tolerant (Rahman et al. 2005) and thermostable (Li and Zhang, 2005) enzymes. Therefore, microbial lipases have been of recent research interests and a number of lipases have been identified, purified and characterized to date.

In general, microbial lipases are 20–60 kDa proteins, with an active Ser residue of the active site structure Ser-His-Asp. Asp may be replaced by Glu in case of *Geotrichum candidum* lipases, which have specificity for hydrolysis of fatty acids with *cis*-unsaturated double bonds. Also, lipases share a consensus sequence of G-X-S-X-G, were X may be any amino acid residue. The lipases belong to the α/β hydrolase family (Ollis et al. 1992) with a central β-sheet, containing the active Ser placed in a loop termed the catalytic elbow. Interfacial activation occurs in presence of a substrate which takes place by the movement of a lid and exposure of the hydrophobic pocket and the active site structure above the critical micellar concentration (CMC) of the substrate (Svendsen, 2000). This interfacial activation is unique to the class of lipases and is also responsible for the versatility of the reactions they catalyse; hydrolysis, esterification, transesterification and interesterification of fats and oils.

The first microbial lipase structure studied was that of the *Rhizomucor miehei* lipase by Brady et al. (1990) from X-ray crystallographic analysis. It showed that this enzyme had an active site triad as that of the serine proteases. X-ray crystallography was and still continues to be a powerful tool for structure determinations of most biological macromolecules. Recently, especially in this millennium, other approaches also have come into practice for structural analyses, including the use of bioinformatics tools for structure predictions up to the tertiary levels of protein organization. In this review, we provide a basic concept of crystallization and X-crystallographic studies of few microbial lipases. Also, various instrumental and chemical methods of structure analysis have been presented and a description of the structures of microbial lipases and their characteristics studied so far has been discussed.

## Crystallization and X-ray Crystallographic Analysis

The fundamental approach in X-ray crystallography is crystallization of the molecule under study. This may seem to be a simple task, but the preparation of good quality crystals is a major limiting step in most cases. Several classical methods of crystallization are in practice (Table 1) and a vast literature is available for ready references; however, efficient methods of growing pure crystals suitable for X-ray diffraction analysis are still to be addressed. McPherson (1990) has reviewed different approaches for crystallization of macromolecules and has also emphasized that macromolecular crystallization is still a poorly understood phenomenon. This review presents a wide analysis of crystallization from supersaturated solutions, growth and properties of crystals, various precipitating agents, factors influencing protein crystal growth and some useful considerations for an efficient crystallization strategy. A contemporary report by Durbin and Feher (1990) describes the mechanisms of crystal growth of proteins by freeze-etch electron microscopy studies, using lysozyme crystals. The report derives that growth occurs by a lattice defect mechanism at low supersaturation and by two-dimensional nucleation at high supersaturation. Abergel et al. (1991) have analysed the systematic use of an Incomplete Factorial approach for design of protein crystallization experiments. The strategy described by Abergel et al. (1991) can aid other experimentalists to design experiments to crystallize their own proteins. However, this approach hinders the X-ray diffraction analysis of lipases that have significant amounts of carbohydrates. Lipase crystals have also been obtained by nucleation and growth from clarified, concentrated fermented broths by bulk crystallization (Jacobsen et al. 1998). A recent crystallization strategy is reported by Wadsten et al. (2006) for membrane proteins by lipidic sponge phase crystallization. However, classical methods such as hanging drop and sitting drop vapor diffusion methods in presence of saturating amounts of ammonium sulphate and/or polyethylene glycol are in common use in day-to-day laboratories.

Lipase structures have been widely investigated by X-ray crystallography in open or closed conformations. X-ray diffraction analyses of a few microbial lipases are briefly described.

The crystal structure of *Rhizomucor miehei* lipase at 1.9 Å resolution using X-ray single crystal diffraction data is reported with refinement of the structure to an R-factor of 0.169 for all available data. Prior to this study, *Rhizomucor miehei* lipase (RmL) complexed with inhibitors were analysed at 3 Å resolution (Brzozowski et al. 1991) and at 2.6 Å resolution (Derewenda et al. 1992a), while, this study presents a detailed analysis of the three-dimensional structure of RmL in its native form (Derewenda et al. 1992b). Lipase I from *Rhizopus niveus* was crystallized by the hanging drop vapor diffusion with cell dimensions of a = b = 83.7 Å, c = 137.9 Å and the diffraction pattern extended to 2.5 Å resolution (Kohno et al. 1993). Lipase crystals from *Staphylococcus hyicus* were obtained using dimethyl sulphoxide (DMSO) and isopropanol, with a = 73.31 Å, b = 77.96 Å and c = 169.81 Å and diffracted to 2.8 Å resolution. (Ransac et al. 1995). Lipase crystals from *Bacillus stearothermophilus* were obtained by hanging drop vapor diffusion method using ammonium sulphate. The unit-cell parameters were a = 118.5 Å, b = 81.23 Å and c = 99.78 Å and diffracted well at 2.2 Å in native form (Sinchaikul et al. 2002). Lipase from *Candida rugosa* was first determined in an open conformation by X-ray crystallography as reported by Grochulski et al. (1993). In 2003, Mancheno et al. have reported the crystal structure of Lipase 2 isoenzyme of *Candida rugosa* at 1.97 Å resolution in its closed conformation. Lipase crystals of *Penicillium expansum* were obtained by the sitting drop vapor diffusion crystallization with unit cell parameters of a = b = 88.09 Å and c = 126.54 Å. Diffraction data were collected to a resolution of 2.08 Å (Bian et al. 2005).

## Instrumental Techniques for Structure Analysis

By and large, X-ray crystallography is the powerful tool for most macromolecular structural elucidations. Of recent interests, other instrumentations have emerged with more sophistications and present valuable tools for protein structure analysis. A few important instrumentation methods are reviewed in view of their current applications.

X-ray crystallography is the oldest and most precise method of structure analysis, in which a beam of X-rays is reflected from evenly spaced planes of a single crystal, producing a diffraction pattern of spots called reflections. Each reflection corresponds to one set of evenly spaced planes within the crystal. The density of electrons within the crystal is determined from the position and brightness of the various reflections observed as the crystal is gradually rotated in the X-ray beam; this density, together with supplementary data, allows the atomic positions to be inferred.

Circular dichroism (CD) has become increasingly recognized for examining the structure of proteins in solution. A significant improvement in the provision of CD instrumentation has occurred in recent years. Kelly et al. (2005) have reported a brief summary of the CD technique and its applications with particular reference to the study of proteins. The important practical aspects of performing CD experiments on proteins have been addressed which provide a clear guidance as to how reliable data can be obtained and interpreted. CD instruments, known as spectropolarimeters measure the difference in absorbance between the L (left) and R (right) circularly polarized components in terms of the ellipticity (θ) in degrees. The CD spectrum is obtained when dichroism is a function of wavelength. A CD spectral analysis serves to understand various structural features of proteins. The secondary structure composition such as % helix, sheet, turns from the peptide bond region, tertiary structure fingerprint, integrity of cofactor binding sites, conformational changes in proteins, protein folding and overall structure features of proteins are all attributed to the study of CD spectra of proteins. Interestingly, an integrated software package for CD spectroscopic data processing, analysis and archiving, known as the *CD tool*, has been developed by Lees et al. (2004). *CD tool* is a multiplatform graphical user interface (GUI) cross-instrument application package, containing a range of features associated with data handling from initial processing to final storage of data and association with related protein data bank (PDB) crystal structure files. Secondary structures of proteins using vacuum-uv CD spectroscopy has also been studied in the case of lipase from *Pseudomonas cepacia* and other globular proteins (Matsuo et al. 2005).

Fourier transform infra red (FTIR) spectroscopy is being increasingly used for investigating protein structure and stability (Haris and Severcan, 1999). Different conformational types result in different absorption bands in a FTIR spectrum, which are usually broad and overlapping. To overcome these, Severcan et al. (2004) have successfully reported the use of artificially generated spectral data to improve protein secondary structure prediction from FTIR spectroscopy.

Mass spectroscopy is a versatile tool for protein analysis and has contributed much to the field of proteomics, in conjunction with two-dimensional electrophoresis. Proteomics helps to define the functions and interrelationships of proteins in an organism. As genome sequence information has accumulated, the paradigm has shifted from sequencing to identification of proteins, which has been facilitated by advances in ionization and mass analysis techniques for mass spectrometry. Electrospray ionization (ESI) and Matrix-assisted laser desorption/ionization (MALDI) methods are currently the principal methods for peptide/protein ionization and have been linked to high-throughput sample preparation techniques. Large-scale protein identification has been made possible using mass spectrometry as reviewed by Lin et al. (2003), with emphasis on its methods and applications. Two-dimensional gel electrophoresis (2DGE) is another important technique for proteome analysis by separation in a first dimension using isoelectric focusing (IEF), and then subjected to SDS-PAGE in the second dimension. A 2DGE is very effective for differential analysis of proteins by separation and visualization, but does not explicitly identify proteins which therefore require a further analytical step for identification or sequencing. Mass spectrometry is now routinely used to identify proteins separated by 2DGE. MALDI-TOF mass spectroscopy is widely used for identification of proteins from 2DGE. Meunier et al. (2005) have investigated data analysis methods for detection of differential protein expression using 2DGE in order to minimize false positives, at the same time, without losing information with false negatives.

Nuclear magnetic resonance (NMR) is another important method in the study of protein structure analysis. Jonas (2002) has reviewed the studies of proteins using high resolution NMR. It is stated that the combination of advanced high resolution NMR technique with high pressure capability is a powerful experimental tool in studies of protein folding. The main advantages of using high resolution and high pressure NMR are the uses of 1-D NMR for determination of structural and dynamic changes in different regions of the protein and the allowance of distance specific information to be obtained between amino acid residues in different regions of the protein.

Electron spin resonance (ESR) spectroscopy in combination with site-directed spin labeling (SDSL) is also an efficient tool for determination of protein structure, dynamics and interactions. Low microwave-amplitude ESR has been used as a novel method especially suitable for studying moderately immobilized spin labels, such as those positioned at exposed sites in a protein (Hedin et al. 2004).

Fluorescence methods are being increasingly used in biochemical characterizations because of its inherent sensitivity. Fluorescence spectroscopy has been employed for monitoring the changes in fluorescence of *Humicola lanuginosa* lipase (HLL), by comparison of the conformations of HLL in an aqueous buffer and dissolved in its substrate, triacetin (Jutila et al. 2004). Triacetin is optically transparent and does not impede the use of fluorescence spectroscopy. It has been revealed that Trp89 plays an important role in the structural stability of HLL, and, the carbohydrate moiety attached to Asn33 has only minor effects on the conformational dynamics of the lid. The analysis of differences in frequencies between the two modes of motion augmented in triacetin, indicated that the motion of the Trp89 side chain becomes distinguishable from the motion of the lid. Thus, steady state and time-resolved fluorescence spectroscopy enabled the analysis and identification of specific structural features of the HLL dissolved in its substrate. Spectroscopic methods have also been applied to analyse the thermal stability of proteins as in the case of *Chromobacterium viscosum* lipase (CVL), whereby, Melo et al. (2000) have studied the CVL thermal stability based on assessment of fluorescence, circular dichroism and static light scattering measurements.

Differential scanning calorimetry revealed unfolding of HLL at 74.4 °C demonstrating significant contribution of Trp residues to the structural stability of the enzyme, when compared with its mutants (Zhu et al. 2001). Small angle X-ray scattering measurements (SAXS) determined the lamellar structure of lipase modified with fatty acids in an aqueous buffer and in n-hexane (Maruyama et al. 2001).

Another major technique is the use of isoelectric focusing electrophoresis to discriminate between closed and open conformations of lipases based on their isoelectric points, as studied by Miled et al. (2005) for HLL and other lipases. They have deduced a significant difference in the isoelectric points between the closed (native) and open (inhibited) conformations, resulting in a distinct electrophoretic pattern, thereby, providing an easy experimental tool for a given lipase.

## Chemical Methods of Structure Analysis

As instrumentation is the basic platform for investigations of protein structures, so are chemical modification strategies of proteins due to their primary, secondary and tertiary structural features, hydrophobicity, similarities of motifs and patterns and other structural arrangements.

Molecular modeling plays a key role in structural biology in interpretations of protein structures with experimental observations. Current modeling methods are extremely useful qualitatively and help to predict increased selectivity of biocatalysts by substrate modification or by site-directed mutagenesis. On the contrary, quantitative predictions are still not reliable, while, modeling is also limited by the availability of three-dimensional structures (Kazlauskas, 2000). Molecular modeling has been applied to *Rhizopus oryzae* lipase (ROL) based on a homology model by Holzwarth et al. (1997) using docking calculations to explain reversals in enantioselectivity. Modeling showed that *sn*-2 substituent binds in a ‘hydrophobic dent’. A flexible β-bond of the substituent avoids clashes with Leu258, but with a rigid β-bond, it can avoid clashes only by turning the substrate so that the *sn*-3 group is in the hydrolysis site. However, modeling has its limitations in prediction of the degree of enantioselectivity.

Electrostatic potential is one of the critical factors explaining lipase/esterase activity based on its distribution on the molecular surface. Other important factors include the presence and distribution of polar and hydrophobic residues in the active cleft. A negative potential in the active site is correlated with maximum activity towards triglycerides (Peterson et al. 2001).

Modifications of lipase with stearic acid or other fatty acids explained the activation mechanism of modified lipases in relation to structure (Maruyama et al. 2001). Shibamoto et al. (2004) have studied the molecular engineering of ROL using a combinatorial protein library constructed on yeast cell surface, thereby, providing a screening method for novel mutant lipase based on yeast cell-surface displayed mutant library. Modification of lid sequence of lipases show that the lid is a structural and a functional determinant of lipase activity and selectivity. This has been observed by Secundo et al. (2006) for *Candida rugosa* (CRL), *Pseudomonas fragi* (PFL) and *Bacillus subtilis* (BSL) lipases. A CRL chimera enzyme obtained by replacing its lid with that of another CRL isoform was found to be affected in both activity and enantioselectivity in organic solvent. Variants of the PFL protein in which three polar lid residues were replaced with amino acids strictly conserved in homologous lipases displayed altered chain length preference profile and increased thermostability. On the other hand, insertion of lid structures from structurally homologous enzymes into BSL, a lipase that naturally did not possess a lid structure, caused a reduction in the enzyme activity and altered substrate specificity. These results strongly support the concept that the lid plays an important role in modulating not only activity, but also specificity, enantioselectivity and stability of lipase enzymes.

Micellar sodium dodecyl sulfate (SDS) is known to stabilize α-helical conformation in peptides derived from helical regions of proteins, although the precise mechanism remains unclear. This effect has been investigated by Montserret et al. (2000) demonstrating that electrostatic interaction play a significant role in the formation and stabilization of SDS-induced structure.

Use of combinatorial design has earlier been applied to the evolution of increased thermostability, in which a diverse library of proteins is generated and screened for variants with increased stability. Current trends are towards the use of data-driven methods that reduce the library size by using available data to choose areas of the protein to target, without specifying the precise changes. Bommarius et al. (2006) have used high-throughput screening methods for enhancement of protein stability by a combination of these methods which lead to the rapid improvement of protein stability for biotechnological purposes.

Antibodies with enzymatic activities are known as abzymes. Of recent interests, Leong et al. (2007) have studied the selection of lipolytic abzymes from the phage displayed antibody libraries against a transition state analog of lipases. This method is presented as an efficient and convenient means to find new abzymes.

## Bioinformatics Approaches for Structure Analysis

**Figure 1:**
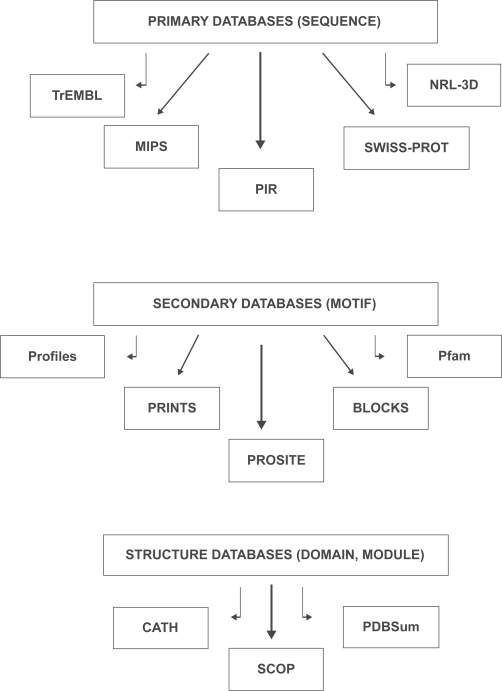
Hierarchy of bioinformatics tools for protein structure analysis.

Structural knowledge is vital for complete understanding of life at the molecular level. An understanding of structure can lead to derivations of functions and mechanisms of action of proteins. From a practical point of view, the sequence-structure gap is a main factor in motivating the need for predictions of protein structure. A hierarchy of the bioinformatics approaches in protein analysis is represented in Figure 1.

Bioinformatics is a novel approach in recent investigations on sequence analysis and structure prediction of proteins. In general, protein sequence databases may be classified as primary and secondary databases, composite protein pattern databases and structure classification databases. Primary and secondary databases are used to address different aspects of sequence analysis, because they store different levels of protein sequence information. Primary databases are the central repositories of protein sequences, while, secondary databases are based on the analysis of sequences of the primary ones (Table 2). Composite protein pattern databases have been emerged with a view to create a unified database of protein families. *ProWeb* (Henikoff et al. 1996) is a dedicated protein family website, providing information about individual families through hyperlinks to existing web resources maintained by researchers in their own fields. Protein structure classification databases have been established based on the structural similarities and common evolutionary origins of proteins. SCOP (Structural classification of proteins, Murzin et al. 1995), CATH (Class, Architecture, Topology and Homology, Orengo et al. 1997) and PDBSum (Laskowski et al. 1997) are the major classification schemes. Thus, bioinformatics tools for protein analysis provide a wealth of information related to sequences and structures of proteins. A number of tools are also available for protein structure visualization (Table 3) and protein identification and characterization (Table 4).

Earlier, protein secondary structure and active site predictions were obtained by alignment of homologous sequences described by Zvelebil et al. (1987). Another study was the analysis and predictions of different β-turns by Wilmot and Thornton (1988). More recently, a new database of aligned protein domains known as DOMO has been developed by Gracy and Argos (1998). DOMO can be accessed through the sequence retrieval system (SRS). A form-based query manager allows retrieval of familial domain alignments by identifiers, sequence accession numbers or keywords. The DOMO sequence analysis provides a simple tool for determining domain arrangements, evolutionary relationships and key amino acid residues in a query protein sequence. With the recent revolutions in bioinformatics, new software tools have been designed to meet updated protein information. Bachinsky et al. (2000) have developed Prot_Pat 1.3, an updated database of patterns to detect local similarities, containing patterns of more than 13,000 groups of related proteins in a format similar to that of PROSITE. Simultaneoulsy, Pleiss et al. (2000) have constructed a database exclusively on lipases to understand and exploit sequence-structure-function relationships. Lipase Engineering Database (LED) serves as a useful tool for protein engineering to help understand the functional role of individual amino acids by reference to annotated aligned sequences and superimposed structures of microbial lipases. The LED is available at http://www.led.uni-stuttgart.de. Of recent interests in bioinformatics for protein structure analysis, Sheehan and Sullivan (2006) have used online resources for homology modeling of milk enzymes. In another report, it has been studied that conformation biases of amino acids play an important role in protein folding, refining domain, structure prediction and structural proteomics, based on the tripeptide microenvironment from PDB (Protein Data Bank) database (Yang et al. 2006). Kartik et al. (2006) have developed a simple web-based computational tool http://www.ccmb.res.in/bioinfo/dsbcp which allows flexible queries to be made on the database in order to retrieve useful information on the disulfide bond containing proteins in the PDB. Thereby, the database may be useful to select suitable protein structure templates in order to model the more distantly related protein homologs/analogs using the comparative modeling methods. Structural bioinformatics has also been applied to structure prediction of membrane-binding cytosolic peripheral proteins (Bhardwaj et al. 2006).

## Structural Features of Lipase

Lipase structural features are important characteristics for protein engineering to provide efficient biocatalysts and make use of their unique structural specificities for commercial exploitation. In this view, many efforts have been attempted to analyse and characterize significant structural data.

A novel structural approach has been devised to distinguish lipases from esterases (Fojan et al. 2000). Lipases have a lid-like structure which is an important entity for exposing a hydrophobic patch in presence of a substrate. The reaction takes place at the oil-water interface by movement of the lid to allow access of the substrate to the catalytic site, while esterases do not display a lid structure. Akoh et al. (2004) have described the unique family of GDSL hydrolases with multifunctional properties. This new subclass of lipolytic enzymes possesses a distinct GDSL sequence motif different from the G-X-S-X-G motif in many lipases. GDSL motif is a consensus amino acid sequence of Gly, Asp, Ser and Leu around the active site Ser.

**Figure 2:**
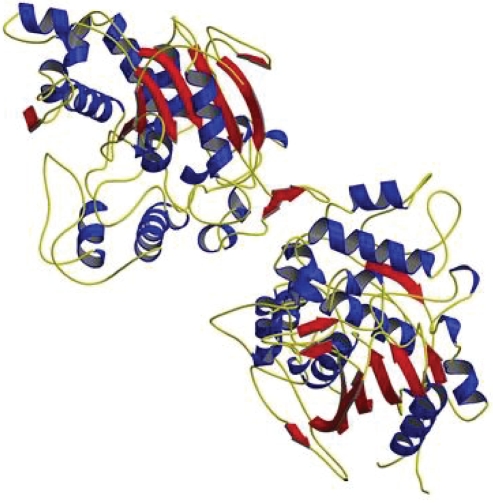
3-D structure of *Bacillus stearothermophilus* (Tyndall et al. 2002).

Various microbial lipases of bacterial and fungal origins have been investigated for analysis of their structures. We summarize the structural properties of different lipases studied in the recent past. The structures of *Pseudomonas* lipases have been well conceived since the last decade. Schrag et al. (1997) have studied the open conformations of *Pseudomonas cepacia* and *Pseudomonas glumae* lipases suggesting that the conformational changes are important for interfacial activation of these bacterial lipases and that the protein conformation depended strongly on the solution conditions, perhaps by the dielectric constant. *Pseudomonas aeruginosa* lipase has a single functional disulfide bond, shown by a shift in electrophoretic mobility after treatment with dithiothreitol (DTT) and iodoacetamide. The structural model predicts a catalytic triad consisting of Ser82, Asp229 and His251, with a disulfide bond between Cys813 and Cys235. Residues Asp38 and Glu46 are located on the surface of the enzyme. A striking prediction was the lack of a lid-like α-helical loop structure covering the active site when the substrate existed either as monomeric solutions or aggregates, confirming the absence of interfacial activation (Jaeger et al. 1993). In other studies of *Pseudomonas* lipases, the role of calcium on the structure and function of a calcium-dependent family 1.3 lipase was characterized and was observed that the C-terminal domain folding was induced by calcium binding (Amada, 2001). In another report, the role of a nine-residue sequence motif in secretion, enzyme activity and protein conformation of a family 1.3 lipase has been described (Kwon et al. 2002). A similar study reported that the repetitive nine-residue sequence motif contributed to the intracellular stability and secretion efficiency of *Pseudomonas* lipases (Angkawidjaja et al. 2005). Prior to these studies, it has been determined by Liebeton et al. (2001) that the disulfide bond in *Pseudomonas aeruginosa* lipase stabilized the lipase structure, although it was not required for interaction with its foldase. The complete amino acid sequence of mono-and diacylglycerol lipase from *Penicillium camembertii* has been determined to consist of 276 amino acid residues with two disulfide linkages and one potential N-glycosylation site (Isobe and Nokihara, 1993). The 3-D structural model of *Bacillus stearothermophilus* P1 revealed a topological organization of the α/β hydrolase fold. The model structure included both α-helix and extended β-sheet secondary structures in the folded protein, and the β-sheet was in the core region surrounding with α-helix. The helix span between Phe180 to Val197 formed the lid of the model lipase. Ser113, Asp317 and His358 formed the catalytic triad (Sinchaikul et al. 2001). Tyndall et al. (2002) have investigated for the first time the 3-D structure of *B. stearothermophilus* P1, as a model for thermostable enzymes, with a unique zinc-binding site which may play a role in enhancing thermal stability (Fig. 2). The lipase from *Bacillus subtilis* showed a single, globular compact domain with dimensions of 35 × 36 × 42 Å. Its fold conformed to the α/β hydrolase fold, although it lacked the β1, β2 strands of the canonical fold. The active site triad consists of Ser78, Asp134 and His157 (Eggert et al. 2002). The primary structure of a novel lipase from *Streptococcus* sp. N1, showed a consensus sequence containing the active serine [VAGHSIGG], a conserved H-G dipeptide in the N-terminus and a potential site for N-linked glycosylation at amino acid residues 129–131 (Tripathi et al. 2004).

Glycosylation is an important feature of eukaryotic lipases, a distinct characteristic of the higher order. Glycosylation is known to contribute to the stability of lipase, but does not affect the enzyme activity (Isobe and Nokihara, 1993). Glycosylation of only a few lipases are reported to due the complexity of its elucidations. However, recently, a number of techniques have evolved to analyse the structural aspects of glycosylation. A strategy for the identification of site-specific glycosylation in glycoproteins using MALDI-TOF mass spectrometry has been described (Mills, 2000). Also, Dell and Morris (2001) have reviewed the various mass spectroscopic techniques as applied for the determination of glycoproteins. More recently, infrared spectroscopy has been used to evaluate the glycosylations of proteins, showing distinct absorption bands for the sugar moiety, the protein amide group and water (Khajehpour et al. 2006). Tang et al. (2001) have reported that glycosylation conferred thermostability to the lipase, while, it did not have any catalytic effect, concluding that glycosylation may effect the structure, stability and movement through the secretory pathways of the lipase.

## A Case-Study: *Candida rugosa* Lipase

*Candida rugosa* lipases (CRL) [exist as isoforms] have been the widely studied lipases in open as well as closed conformations. CRL are of immense significance due to potential applications of commercial interests and have been well-documented as efficient biocatalysts for biotransformations (de Maria et al. 2006). CRL consists of 534 amino acid residue polypeptide chain, with a predicted molecular mass of 60 kDa (Mancheno et al. 2003), showing an α/β hydrolase structure, with a catalytic triad (Ser209-Glu341-His449) and a lid that covers the active site (Akoh et al. 2004). The active site in CRL is covered by an α-helical structure (residues 65–94), composed of variable amino acidic composition of the lid (de Maria et al. 2006). The lid structure is fixed by a disulphide bond (Cys60-Cys97) and an ionic interaction between Glu96 and Arg37 (Cygler and Schrag, 1999). Lipases also contain the consensus sequence G-X-S-X-G, where, X = any amino acid residue (Svendsen, 2000). Distinct phenylalanyl-rich region and an aliphatic-rich region have been revealed by structural comparisons of lipase 2 at 1.97 Å resolution in its closed conformation. The aliphatic-rich region is identical to other isoforms, while, the phenylalanyl content is specific for each lipase isoforms, responsible for their varied lipase/esterase characteristics (Mancheno et al. 2003).

## Conclusions

In the light of discussion on important instrumental, chemical and bioinformatics approaches, we highlight the basic strategies for structural elucidations of proteins, as in the case of microbial lipases. This review also encompasses the recent advancements in protein science and research for structure analysis. *In toto*, it is attempted to describe a better understanding of the structural characteristics of proteins with evidences of lipase structural features.

## Figures and Tables

**Table 1:** Protein crystallization methods.

**Methods of crystallization**	**Subtypes**
Batch	Batch; Microbatch
Seeding	Macroseeding; Microseeding
Nucleation to support growth only	Free interface diffusion; local nucleation
Vapor diffusion	Hanging drop; Sitting drop
Dialysis	-
Lipidic sponge phase crystallization (for membrane proteins)	-

**Table 2:** Primary and Secondary databases for protein analysis.

**Type of database**	**Databases**	**Targets**	**Web address (URL)**
Primary	PIR	Sequence	http://pir.georgetown.edu
	MIPS	Sequence	http://mips.gsf.de
	Swiss-Prot	Sequence	http://www.ebi.ac.uk/swissprot/
Secondary	PROSITE	Patterns	http://www.expasy.ch/prosite/
	PRINTS	Fingerprints	http://www.bioinf.manchester.ac.uk/dbbrowser/PRINTS/
	Pfam	^a^HMMs	http://pfam.sanger.ac.uk/
		^b^MSA	
	BLOCKS	Motifs	http://blocks.fhcrc.org/

a-Hidden Markov Models.

b-Multiple sequence alignments.

**Table 3:** Protein visualization programs.

**Program**	**Function**
RasMol	3-dimensional visualization
Cn3D	3-dimensional visualization, linked to sequence alignments
Chime	3-dimensional visualization
TOPS	Visualization of protein folding topologies
DSSP	Finds secondary structure elements in an input structure
Surfnet	Visualization of protein surface
PROCHECK	Checks stereochemical quality of protein structures
PROMOTIF	Analyses protein structural motifs

**Table 4:** Protein identification and characterization programs.

**Program**	**Function**
AACompIdent	Identification of amino acid composition
TagIdent	Identification of proteins using mass spectrometric data
PeptIdent	Identification of proteins using peptide mass fingerprinting data
MultiIdent	Identification of proteins using pI, MW, amino acid composition
Propsearch	Find putative protein family
PepSea	Identification of protein by peptide mapping or peptide sequencing
FindPept	Identification of peptides resulting from unspecific cleavage of proteins
TMAP; TMHMM	Prediction of transmembrane helices
ProtParam	Computation of physical and chemical parameters of a protein
